# Both Acidic pH Value and Binding Interactions of Tartaric Acid With α-Glucosidase Cause the Enzyme Inhibition: The Mechanism in α-Glucosidase Inhibition of Four Caffeic and Tartaric Acid Derivates

**DOI:** 10.3389/fnut.2021.766756

**Published:** 2021-10-07

**Authors:** Wenyue Li, Yi Song, Wanshu Sun, Xi Yang, Xuebo Liu, Lijun Sun

**Affiliations:** College of Food Science and Engineering, Northwest A&F University, Xianyang, China

**Keywords:** α-glucosidase inhibition, mechanism, tartaric acid, binding interactions, acidic pH value

## Abstract

The inhibition mechanism of four caffeic and tartaric acid derivates, including caffeic acid (CA), tartaric acid (TA), caftaric acid (CFA) and chicoric acid (CHA) against α-glucosidase was characterized by substrate depletion, fluorescence quenching, isothermal titration calorimetry (ITC) and molecular docking. TA and CA were found with the highest and no inhibition effect respectively, and caffeoyl substitution at 2 and/or 3-OH of TA significantly decreased its inhibition. The enzyme inhibition effects of organic acids were not in an inhibitor concentration-dependent mode, and there was a rush increase in inhibition at a respective acidic pH value, especially for CFA and CHA, suggesting the important role of acidic pH in the enzyme inhibition for both compounds. Besides, CA, CFA and CHA were shown with strong quenching effects on α-glucosidase fluorescence because of π-conjugations between aromatic ring of caffeoyl moiety and that of enzyme fluorescent residues. However, no fluorescence quenching effect was observed for TA due to lack of aromatic ring. Additionally, a direct binding interaction behavior was observed for TA with α-glucosidase according to the fitted independent binding model in ITC, but not for CFA and CHA. Therefore, both acidic pH and binding interactions of TA with α-glucosidase resulted in the enzyme inhibition.

## Introduction

Postprandial hyperglycaemia has been considered as one essential factor inducing disorder symptoms of carbohydrate metabolisms (1). Starch is one primary component of main foods for human beings. The velocity and extent of starch digestion decide blood sugar level after meal to a large extent (2). After starchy foods ingestion, starch is initially digested by salivary and pancreatic α-amylase, producing maltose, maltotriose, maltooligosaccharides and limit dextrin, and then the reducing sugars are further hydrolyzed by α-glucosidase at intestinal brush borders, including maltase-glucoamylase and sucrase-isomaltase, producing glucose that is adsorbed into portal blood through glucose transporters (3). Therefore, inhibiting carbohydrate-hydrolyzing enzymes has been reported to potentially regulate starch digestion and thus control blood glucose level (4).

Some pharmaceuticals have been prescribed to type II diabetes patients to retard the increase in blood sugar content, like acarbose, voglibose, etc., due to the inhibiting effects of these medicines against α-glucosidase (5). However, long-term administration of the prescribed pharmaceuticals may lead to some side-effects, such as flatulence, stomachache, diarrhea (6); therefore, it is necessary to explore and develop natural products that possess a relatively strong inhibitory activity and less side-effects. In recent years, dietary polyphenols have been suggested as one kind of natural inhibitors of α-glucosidase, like tea polyphenols, flavonoids and edible plant phenolic extracts (7). There are structure-activity relationships regarding α-glucosidase inhibition of polyphenols (8). As for flavonoids (one kind of polyphenols with C3-C6-C3 skeleton structures), the hydroxyl groups (-OH), especially that at 3- position of ring C and 5'-position at ring B play an important role in hydrogen bondings of myricetins with the active site of α-glucosidase and thus in the enzyme inhibition (9). The double bonds C2 = C3 can form a conjugation system with C4 = O, which further conjugates with ring A. This promotes the electron delocalization within ring A and C, decreasing the molecular internal energy of myricetin and quercetin, and thus makes the π-stacking of the flavonoids with the enzyme more stable (9, 10). Besides, some structural moieties are also essential in α-glucosidase inhibition of polyphenols, for instance, galloyl moiety (11). The presence of galloyl moiety has been reported to enhance the inhibitory activity of tea polyphenols (catechins and theaflavins) against α-glucosidase by increasing the polyphenol-enzyme binding interactions. This is attributed to the fact that the three -OHs can form hydrogen bondings with the enzyme active site, and that the benzene ring can form π-stacking with the aromatic ring(s) of fluorescent amino acids of the enzyme (12). It is necessary to explore more polyphenol structure-inhibitory relationships in order to increase the efficiency in discovery of natural inhibitors of α-glucosidase.

**Figure 1:**
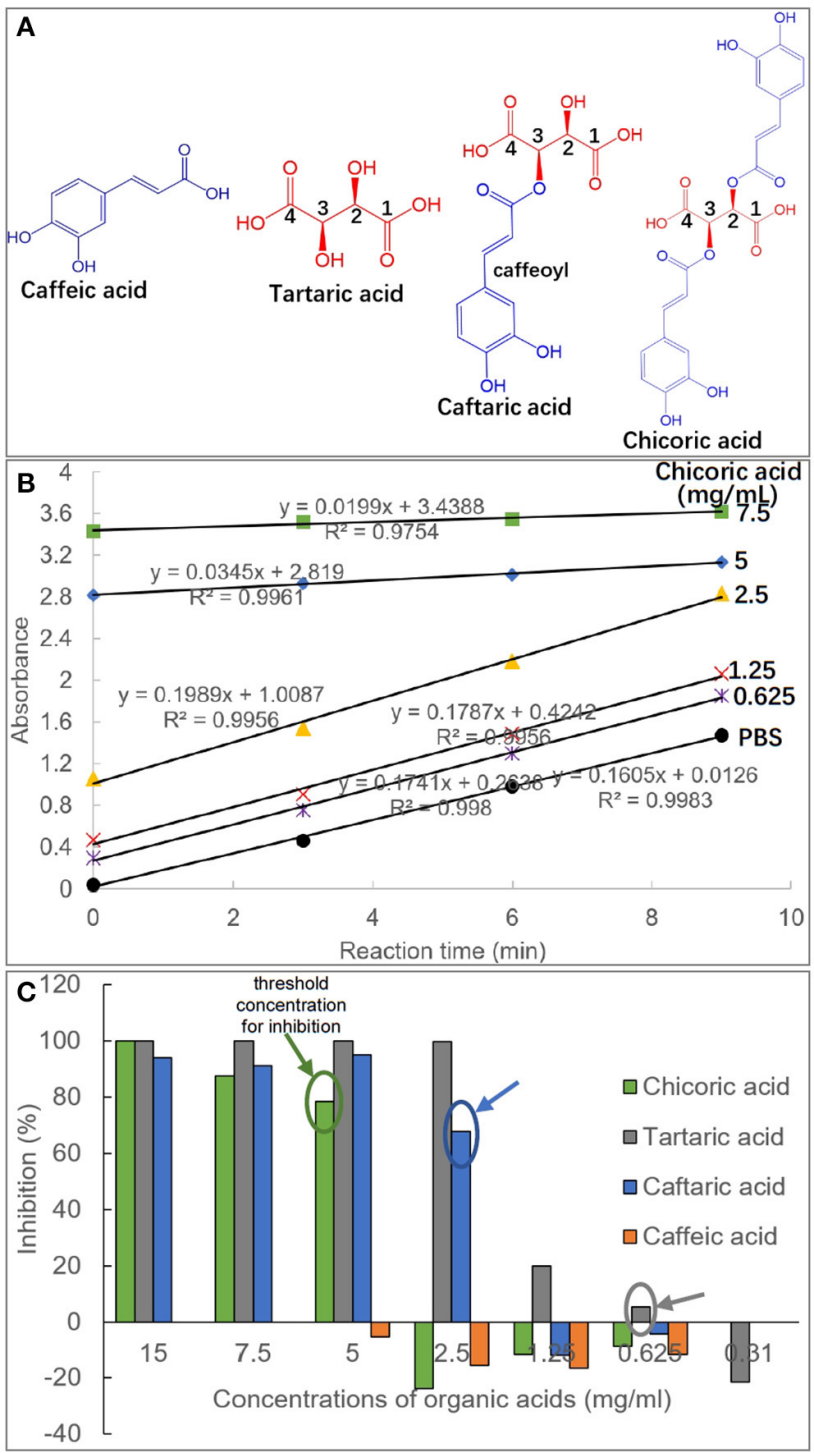
Molecular structures of caffeic acid, tartaric acid, caftaric acid and chicoric acid, and the caffeic acid part in caftaric acid and chicoric acid is defined as caffeoyl moiety **(A)**. The plot of absorbance values of *p*NPG hydrolysis solution against reaction time (min) in the absence and presence of organic acids with different concentrations (taking chicoric acid as an example, and the plots for other organic acids were shown with similar profiles, except for caffeic acid due to its weak inhibitory activity) **(B)**. The initial reaction velocity (*v*) was calculated as the slope of the plot, and the enzyme inhibition effects (%) were obtained using equation (1) for the organic acids at a series of inhibitor concentrations **(C)**. Besides, the threshold concentrations of organic acids for the enzyme inhibition were labeled.

Caffeic and tartaric acid derivates, including caffeic acid, tartaric acid, caftaric acid (one caffeoyl substituted tartaric acid) and chicoric acid (two caffeoyls substituted tartaric acid) (the molecular structures of four compounds shown in Figure 1A) are the predominant organic/phenolic acids existing in green coffee bean, grape and chicory (13, 14). The inhibition of these compounds against α-amylase has been reported and it is found that the caffeoyl moiety is able to enter into and interact with the active site of the enzyme, thus enhancing the competitive inhibition of the organic acids (15). As introduced above, both α-amylase and α-glucosidase are key enzymes for starch digestion. The inhibitory activity of the caffeic and tartaric acid derivates against α-glucosidase, however, has not been studied. Therefore, the enzyme inhibition of the four organic acids and the inhibition mechanism are explored by use of substrate hydrolyzation, fluorescence quenching, isothermal titration calorimetry and molecular docking, revealing how they develop the inhibiting effects and the contribution of structural caffeoyl moiety(s) to α-glucosidase inhibition of caffeic and tartaric acid derivates.

## Materials and Methods

### Materials and Chemicals

α-Glucosidase from *Saccharomyces cerevisiae* (G0660-750UN) and phosphate buffered saline (PBS) tablets were purchased from Sigma-Aldrich Co. (St. Louis, US). *p*-Nitrophenyl-α-_D_-glucopyranoside (*p*NPG) and four organic acids, including caffeic acid, tartaric acid, caftaric acid and chicoric acid were obtained from Yuanye Biotech. Co. (Shanghai, China). Other chemicals in this study were of analytical grade.

### Inhibition of Organic Acids Against α-Glucosidase

The inhibition effects of four organic acids were characterized by determining the initial velocity of *p*NPG hydrolyzation in the absence and presence of the inhibitors according to previous studies (11, 16). The initial velocity was obtained from the slope of the plot of *p*NP equivalent (Δabsorbance value) against the reaction time (min), and the inhibition (*I*) was calculated using the equation (1) as follows (17):


(1) $$\begin{array}{l} I\;\left(\%\right)=\left(1-\frac{v}{v_{0}}\right)\times 100 \end{array}$$

where, *v* and *v*_0_ are the initial hydrolyzation velocity in the presence and absence of organic acids, respectively.

### Determination of pH Values of Organic Acids

The organic acids were dissolved in PBS or 20% DMSO (in PBS) with a series of concentrations same as that in the inhibition determination. Then, 300 μL of each organic acid solution was withdrawn for determination of pH value by use of an INESA pH meter (PHSJ-3F, Shanghai, China) equipped with a BestLab Semi-Micro electrode probe. During pH determination process, the liquid level was kept stable to make the determined values precise.

### Fluorescence Quenching

The fluorescence spectra of α-glucosidase in the absence and presence of organic acids were determined using a Shimadzu® RF-6000 spectrofluorometer according to one previous study (12). The fluorescence quenching constant, *K*_FQ_ was calculated from the Stern-Volmer equation as follows (18, 19):


(2) $$\begin{array}{l} \frac{F_{0}}{F}=1+k_{q}\tau_{0}\left[Q\right]=1+K_{FQ}\left[Q\right] \end{array}$$

where, *F*_0_ and *F* are the maximum fluorescence intensity values in the absence and presence of organic acids. *k*_*q*_ is the bimolecular quenching constant; τ_0_ is the lifetime of fluorophore, and for α-glucosidase the value is 10^−8^ s; [*Q*] is the organic acid concentration.

Additionally, there may be some positive deviations of the Stern-Volmer equation for some quenchers, which causes the fitted plot concave to *y* axis. For this case, the modified exponential form of Stern-Volmer equation is applied as follows (20, 21):


(3) $$\begin{array}{l} \frac{F_{0}}{F}=e^{\left(K_{FQ}\left[Q\right]\right)} \end{array}$$

### Isothermal Titration Calorimetry

The enthalpy changes caused by binding interactions of organic acids with α-glucosidase were determined using a TA® isothermal titration calorimetry instrument (NanoITC, US) according to one previous study (22). Specifically, 50 μL of 5 mg/mL each organic acid that was loaded in an ITC syringe was titrated drop-by-drop into 170 μL of 1.5 mg/mL α-glucosidase solution in an ITC sample cell. The total injection number was 25 with each injection volume of 2 μL. The duration time between each injection was 180 s. The temperature during the titration process was maintained at 25°C with a magnetic stirring at 250 rpm. The titration of organic acid to PBS buffer was applied as the control, and the enthalpy changes of the control was subtracted from that of titration of organic acid to α-glucosidase solution (23). The raw data was obtained as a plot of heat rate (μJ/s) against time (s). Then, it was processed by integration of peak-by-peak and normalization, obtaining a plot of corrected enthalpy per mole of organic acid injection (kJ/mol) against injection order. In the following step, the integrated enthalpy was fitted using an independent (single-site) binding model at the modified and available range of molar ratios of organic acid to α-glucosidase (making the model fitted better and the related constants calculated). The fitting equation (4) of independent binding model is described as follows (24):


(4) $$\begin{array}{l} Q_{i}=\frac{n[M]\Delta H_{itc}V_{0}}{2}\{1+\frac{\left[P\right]}{n\left[M\right]}+\frac{K_{d}}{n[M]}-\\ \sqrt{{\left(1+\frac{\left[P\right]}{n\left[M\right]}+\frac{K_{d}}{n[M]}\right)}^{2}-4\frac{\left[P\right]}{n[M]}}\} \end{array}$$

where, *Q*_*i*_ is the total heat released after injection *i*; *V*_0_ is the volume of the ITC sample cell (here 170 μL); [*M*] is the total concentration of α-glucosidase; [*P*] is the total concentration of each organic acid; *n* is the molar ratios of binding species; Δ*H*_itc_ is the enthalpy change; *K*_*d*_ is the equilibrium dissociation constant of organic acid-glucosidase complex.

### Molecular Docking

A Sybyl 2.0 molecular docking software was used to predict the interaction forces and sites of for binding of organic acids with α-glucosidase (25). The crystal structure of α-glucosidase was obtained from the Protein Data Bank (PDB ID: 3A4A). Notably, to obtain the optimal docking result, the spatial conformations of organic acids were modified and adapted to the docking sites. The binding energy, *E*_b_ was calculated based on the equation (5) as follows:


(5) $$\begin{array}{l} E_{b}=RT\log_{e}\left(10^{-pk_{d}}\right) \end{array}$$

where, *pk*_d_ is the affinity score according to the Surflex scoring function, and the *RT* is 0.59 kcal/mol.

### Statistical Analysis

One-way analysis of variance (ANOVA) followed by Tukey's test (Graphpad Prism 6) was applied to analyze the significant difference between the constants. When *P* < 0.05, the data is considered as statistically significant and thus marked with different superscripts.

## Results and Discussion

### Inhibition of Organic Acids Against α-Glucosidase

The inhibition of four organic acids against α-glucosidase at a series of inhibitor concentrations was studied by determination of initial reaction velocity (*v*) of substrate *p*NPG depletion in the absence and presence of organic acids (Figure 1B). It was found that there was a satisfactory (all *R*^2^ > 0.99) linear correlation between the product amounts (reaction solution absorbance equivalents) and the reaction time (Figure 1B). Therefore, the obtained *v* from the slop of product amount-reaction time correlation was able to indicate the residual activity of α-glucosidase, and thus the enzyme inhibition was calculated using the equation (1). Specifically, CA hardly inhibited the enzyme even at a relatively high concentration (5 mg/mL), at which the other three organic acids were shown with a strong inhibiting effect (Figure 1C), indicating that CA was a very weak inhibitor of α-glucosidase. TA was always shown with the highest inhibition in the four compounds at each organic acid concentrations (Figure 1C).

Besides, the threshold concentration of TA for the enzyme inhibition (the lowest concentration required for inhibition) was determined as 0.625 mg/mL, and the values of CFA (one caffeoyl substituted TA) and CHA (two caffeoyls substituted TA) were 2.5 mg/mL and 5 mg/mL, respectively (Figure 1C). This indicates that caffeoyl substitution at 2-OH and/or 3-OH of TA gradually decreased the inhibitory activity of the organic acid against α-glucosidase. Interestingly, it was found that α-glucosidase inhibition of the four organic acids was not typically inhibitor concentration dependent, that is, there existed a large gap of inhibition effects between the adjacent gradient concentrations of each organic acid. For instance, the inhibition ratios of TA at 1.25 and 2.5 mg/mL were 19.9 and 99.8%, and similarly, 0 and 67.9% of inhibition corresponded to the two concentrations of CFA (Figure 1C). This resulted in the fact that the IC_50_ values of the four inhibitors were not detectable because an inhibitor concentration-dependent inhibition mode is required to obtain this inhibitory constant (4, 26).

It is accepted that inhibition of a polyphenol against α-glucosidase results from binding interactions between them (4). The special inhibition character of the studied organic acids suggests that there may exist additional force (along with organic acid-enzyme binding) that caused α-glucosidase inhibition of these compounds, which was further explored and discussed as follows.

### The Relationships Between Organic Acid pH Values and α-Glucosidase Inhibition

**Figure 2:**
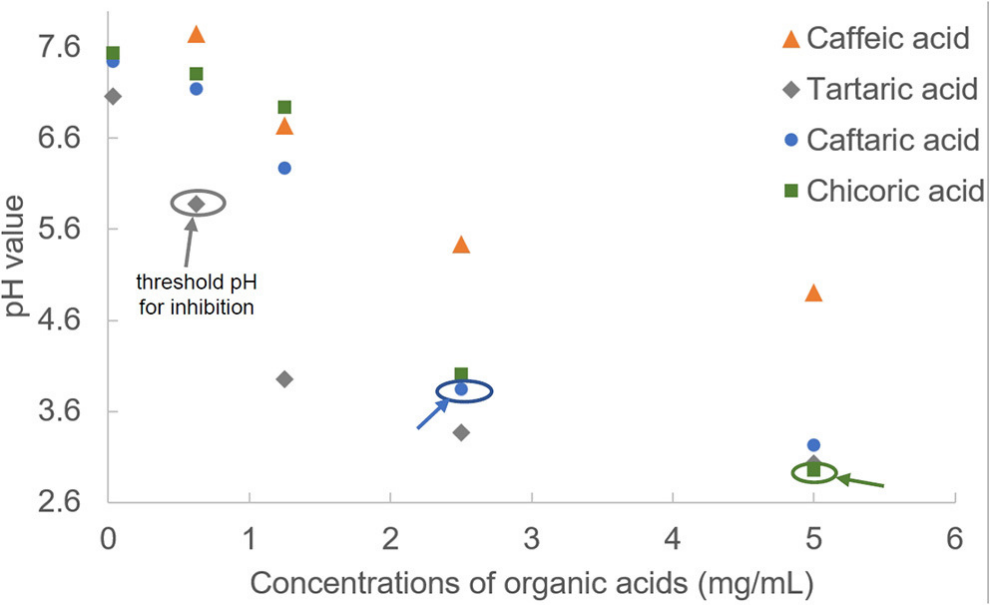
The pH values of organic acids at a series of concentrations. The threshold pH values of organic acids for α-glucosidase inhibition were labeled.

To explore the factors causing the enzyme inhibition of organic acids, the pH values were determined at a series of compound concentrations (Figure 2). Although these organic acids were dissolved in PBS buffer (pH = 7.4), the compound solutions still presented with acidic property that was attributed to the dissociation of carboxylate acid moieties, and all the pH values decreased with the organic acid concentration increasing (Figure 2). It was found that TA and CA were always shown with the lowest and highest pH values at each organic acid concentrations (Figure 2), corresponding to the highest and lowest inhibition effects of the two compounds (Figure 1C).

The large gaps of α-glucosidase inhibition between adjacent pH values for organic acids (from 19.93% at pH 3.95 to 99.8% at pH 3.37 for TA; from 0 at pH 6.27 to 67.9% at pH 3.84 for CFA; from 0 at pH 4.01 to 78.5% at pH 2.95 for CHA, Figures 1C, 2) indicate that the acidic pH of solutions played a crucial role in the enzyme inhibition of organic acids, especially for CFA and CHA as there was no inhibiting effects at the near-neutral pH values for both compounds but a rush increase in inhibition at the obvious acidic pH values (Figures 1C, 2). Usually, an enzyme develops a satisfactory catalytic efficiency at the suitable pH ranges in solutions (27). The optimum pH value for α-glucosidase used in this study (from *Saccharomyces cerevisiae*) is 6.8 (as described in the manufacture instruction). The addition of organic acids caused the significant shift away from this value. Besides, there was hardly any enzyme activity observed when the pH value was below 3, especially for TA and CFA (two compounds with the lowest pH values and the highest inhibition effects) (Figures 1C, 2). Therefore, the decreased catalytic capacity caused by organic acid addition resulted from the acid-oriented denaturation of the enzyme to a large extent that finally caused the enzyme inactivation at a high inhibitor concentration. This result is different from one previous finding that the four organic acids studied, except for CA, caused the reversible inhibition against α-amylase at the available concentrations (15). That is, the acidic pH of the four organic acids contributed less to α-amylase inhibition. This is supposed to be attributed to the fact that the difference in enzymic spatial structures leads to the difference in acid stability/tolerance of the enzymes (28).

However, similar to the analysis of threshold concentration for α-glucosidase inhibition, the threshold pH values of TA, CFA and CHA for the enzyme inhibition were 5.88, 3.84, and 2.95, respectively (Figures 1C, 2). The difference in threshold pH values indicates that acidic pH of organic acid solution was not the only one factor that caused the enzyme inhibition. By this way, the binding interactions between the organic acids and α-glucosidase were further studied by fluorescence quenching, isothermal titration calorimetry and molecular docking approaches as follows.

### Fluorescence Quenching

**Figure 3:**
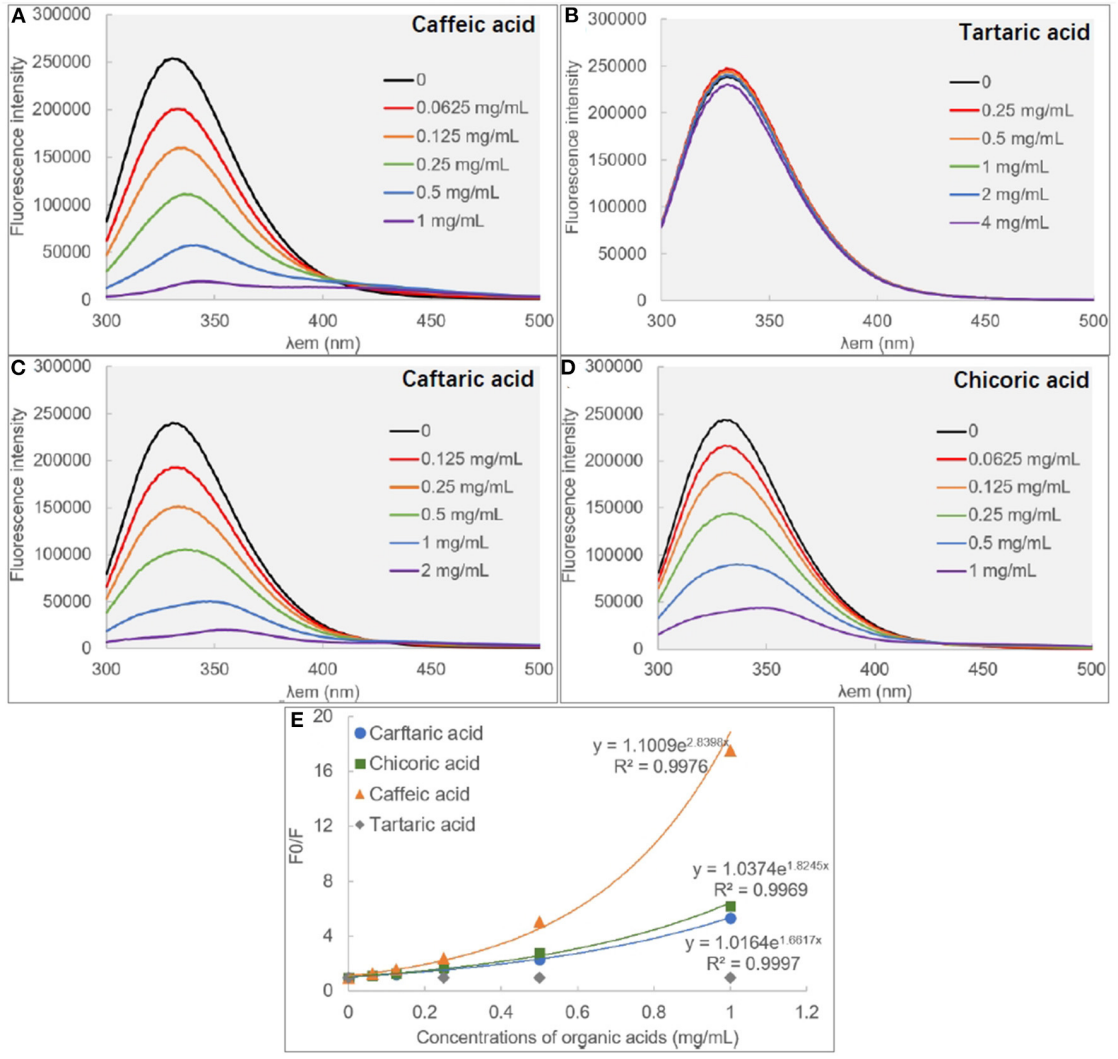
The fluorescence spectra of α-glucosidase in the absence and presence of caffeic acid **(A)**, tartaric acid **(B)**, caffeic acid **(C)** and chicoric acid **(D)**, respectively, and the modified Stern-Volmer equation **(E)** was applied for the quenching analysis.

There are some aromatic amino acids at the active site of α-glucosidase, like Tyr, Trp and Phe, making the enzyme emit fluorescent spectrum at certain excitation wavelengths of ultraviolet light (11, 29). Binding interactions between an exogenous molecule (usually called as a quencher) and the enzyme, especially π-π hydrophobic conjugations between the aromatic ring(s) of the quencher and that of the enzyme, would cause the decrease in the enzyme fluorescence intensity because the π-stacking is able to “cover” the fluorescent property (9). Therefore, the fluorescence quenching approach was used to study the binding interactions between organic acids and α-glucosidase (30) (Figure 3). Interestingly, it was found that although CA hardly showed the inhibitory activity against α-glucosidase, the phenolic acid significantly quenched the enzyme fluorescence (Figure 3A). Similarly, TA was shown with the highest inhibition effect, however, it hardly showed the quenching effect (Figure 3B). Besides, the fluorescence quenching constant (*K*_FQ_) that quantificationally indicates the quenching intensity was also calculated (Table 1), and a higher *K*_FQ_ suggests a higher quenching effect. Therefore, the fluorescence quenching effects of four organic acids followed the order of CHA > CFA = CA >> TA (Table 1), which is different from the order of inhibition effects (TA > CFA > CHA > CA) (Figure 1C). The inconsistency between the inhibition and quenching effect lies in the fact that there is caffeoyl moiety(s) in CA, CFA and CHA (Figure 1A). The benzene (aromatic) ring of the moiety was considered to form π-conjugation with the aromatic fluorescent residues (like Tyr, Phe) of α-glucosidase (which was also indicated by the molecular docking results and discussed as follows), causing the decrease in fluorescent intensity of the enzyme fluorophores (Figures 3A,C,D). By this way, the higher quenching effect of CHA resulted from the more caffeoyl moieties (i.e., the more aromatic rings) in its molecule, and the very weak (hardly any) quenching effect of TA was attributed to the lack of aromatic ring. Therefore, it is concluded that the quenching effect of an organic acid on α-glucosidase fluorescence is highly related with the presence of aromatic ring(s) in the quencher molecule, instead of its inhibitory activity.

**Table 1:** The constants that indicate the inhibitory activity and binding of organic acids with α-glucosidase.

**Organic acids**	${\text{IC}}_{50}^{*}$ **(mg/mL)**	***K*_**FQ**_ (L/mol)**	***k*_**q**_ (10^**10**^ L/(mol·s))**	***K*_**itc**_ (10^**5**^ L/mol)**	***ΔH* (kJ/mol)**	***ΔS* (J/mol·K)**	***n***	***E*_**b**_ (kcal/mol)**
Caffeic acid	>5	511.59^a^	5.12^a^	-	-	-	-	−7.174
Tartaric acid	1.25–2.5^#^	-	-	9.41	−1.216	110.3	360	−7.482
Caftaric acid	1.25–2.5^#^	518.83^a^	5.19^a^	-	-	-	-	−12.841
Chicoric acid	2.5–5.0	865.49^b^	8.65^b^	-	-	-	-	−15.384

* *The IC_50_ values were not able to be calculated because of the rush increase in the inhibition effects at the condition of acidic pH values of organic acids, and the values were estimated according to the inhibition effects at the corresponding organic acid concentrations*.

# *Although the estimated IC_50_ values of tartaric acid and caffeic acid were the same, the inhibitory activity of tartaric acid was higher than caftaric acid*.

*‘-’ means not detectable or calculated due to very weak fluorescence quenching or binding interactions*.

*The different superscript letters in the same column indicate the significantly different (P < 0.05) with each other*.

In addition, the exponential Stern-Volmer equations of CA, CAF and CHA (Figure 3E) indicate that the three organic acids quenched the fluorescence of α-glucosidase through both static and dynamic mechanisms or there was a “sphere-of-action” between organic acids and α-glucosidase (18, 31). To confirm this, the bimolecular quenching constants (*k*_q_) were calculated (Table 1). For a typical dynamic quenching action (collision mechanism), the *k*_q_ value is close to 11 × 0^10^ (18). The *k*_q_ values of three organic acids were around 5–8 times as this value, indicating that there tended to form an interaction sphere region between the quenchers and the enzyme, i.e., apparent static quenching mechanism. In this region, the binding affinity of organic acids to α-glucosidase was stronger than the molecular collision mechanism (dynamic one) and weaker than the complexation mechanism (static one), corresponding to the relatively weak inhibition effects at the low organic acid concentration ranges.

### Isothermal Titration Calorimetry

**Figure 4:**
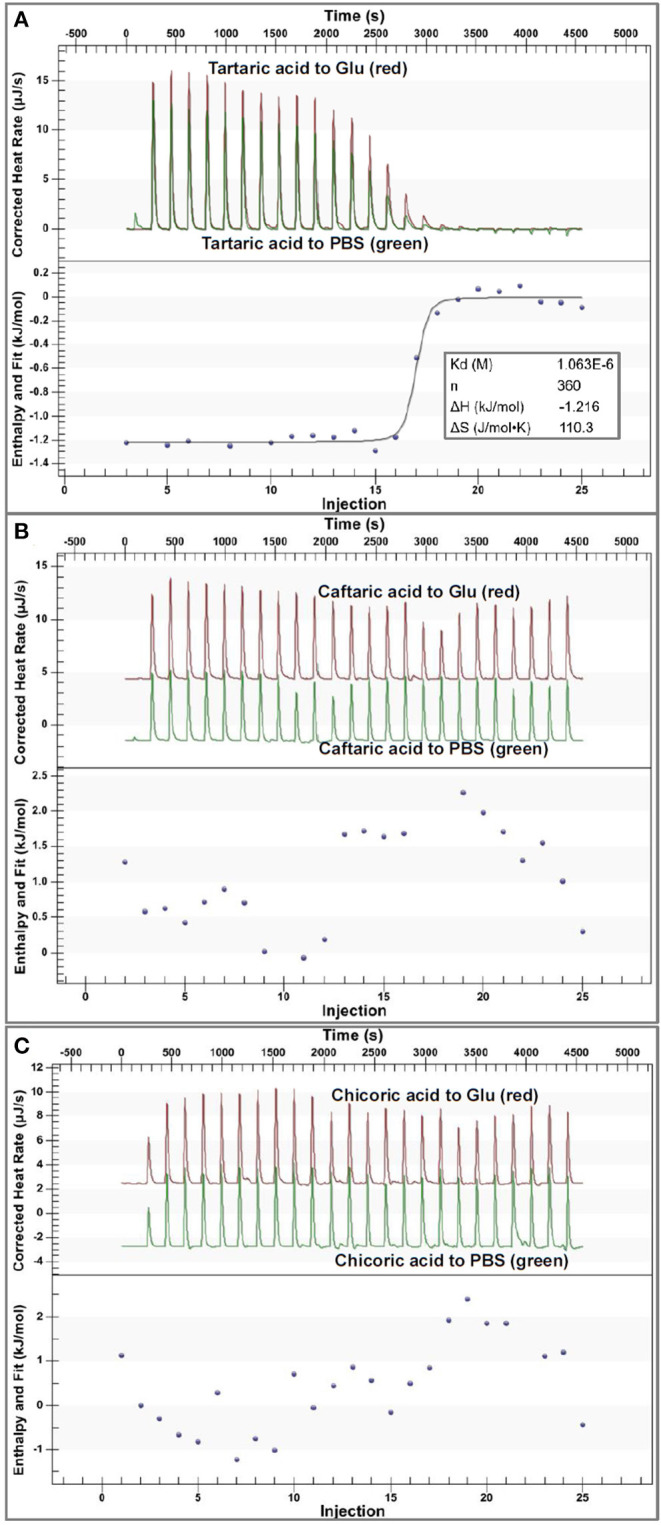
The ITC data for binding interactions of tartaric acid **(A)**, caftaric acid **(B)** and chicoric acid **(C)** with α-glucosidase. In each figure, the upper section indicates the heat flow for titration of organic acids to α-glucosidase solution (the read peak curves) and the heat flow for titration of organic acids to buffer (the green peak curves), and the lower section of **(A)** indicates the fitted plot of the enthalpy changes against injection order using an independent binding model. The input molar ratio of tartaric acid to α-glucosidase was modified within the available range to make the model fit the curve better. However, the plots in **(B,C)** could not be fitted using the independent binding model because of the weak binding interactions of caftaric acid and chicoric acid with α-glucosidase. Notably, the molecular weight of α-glucosidase was estimated during the data analysis, which may cause the difference in the fitted and calculated ITC results, but it was kept consistent in this study.

To directly characterize the binding affinity of organic acids to α-glucosidase, isothermal titration calorimetry (ITC) was applied to analyze the enthalpy changes caused by binding interactions between organic acids and the enzyme (Figure 4). It was found that the titration of organic acids to PBS also caused enthalpy changes (Figure 4) due to the heat of dilution of the ligands from an ITC syringe to a sample cell. After subtracting the released dilution heat, the enthalpy changes that resulted from organic acid-glucosidase binding interactions were obtained for each titration. Then, the correlations of enthalpy changes against injection order were fitted using the independent (single-site) binding model.

Specifically, the corrected enthalpy changes during the titration of TA to α-glucosidase was observed as negative (Figure 4A), indicating that the ligand-enzyme binding process was an exothermal one (23, 32). This is consistent with the general heat-releasing character for binding of a biomicromolecule with a protein in previous studies (23, 32, 33). Besides, the well-fitted independent binding model (Figure 4A) suggests that the binding sites of α-glucosidase with TA tended to be specific or homogenized (which may be the active site of the enzyme). From the fitted binding model equation, the dissociation constant of TA-glucosidase (*K*_d_) was obtained (Figure 4A); therefore, the association constant between the two molecules (*K*_itc_) that indicates the binding affinity of TA to the enzyme was calculated as the reciprocal of *K*_d_ (1/*K*_d_, Table 1) (34). The stoichiometry, *n* that means the molar ratio of ligand to enzyme is also able to be obtained from the binding model equation, and a higher *n* indicates more ligand molecules are required to occupy the binding sites of the enzyme (33). In this study, the *n* value for TA- glucosidase binding was determined as 360 (Table 1), which was considered as relatively high compared with some other micromolecule-enzyme binding interactions, like EGCG-amylase and ellagitannin-bovine serum albumin (22, 33). This may be caused by the relatively weaker TA-glucosidase binding interactions than the above ones. Also, it is supposed to arise from the lower molecular weight of TA, which caused the smaller number of non-covalent bondings of one TA molecule with α-glucosidase, and thus more TA molecules were required to saturate the enzymic binding sites. As a result, the entropy value after titration of TA to α-glucosidase was significantly increased, indicated by a large positive entropy change (Δ*S*, Table 1), because the introduction of abundant TA molecules into the enzyme system led to the increase in the disorder degree of the interaction mixture (35).

Additionally, in consideration of the sharp increase in α-glucosidase inhibition along with the pH reduction (Figures 1C, 2), the enzyme inhibition of TA was caused by both the acidic pH and the TA-enzyme binding interactions. On the other hand, the irregular enthalpy changes for the corrected titration of CFA and CHA to α-glucosidase (Figures 4B,C) indicate that the binding interactions of both organic acids with the enzyme were weak or not detectable. By this way, the enzyme inhibition effects of CFA and CHA were mainly attributed to the acidic pH of the phenolic acid/α-glucosidase mixtures. Besides, taking the molecular structures of TA, CFA and CHA into account, caffeoyl substitution at 2 and/or 3-OH of TA decreased the binding affinity of TA to α-glucosidase.

### Molecular Docking

**Figure 5:**
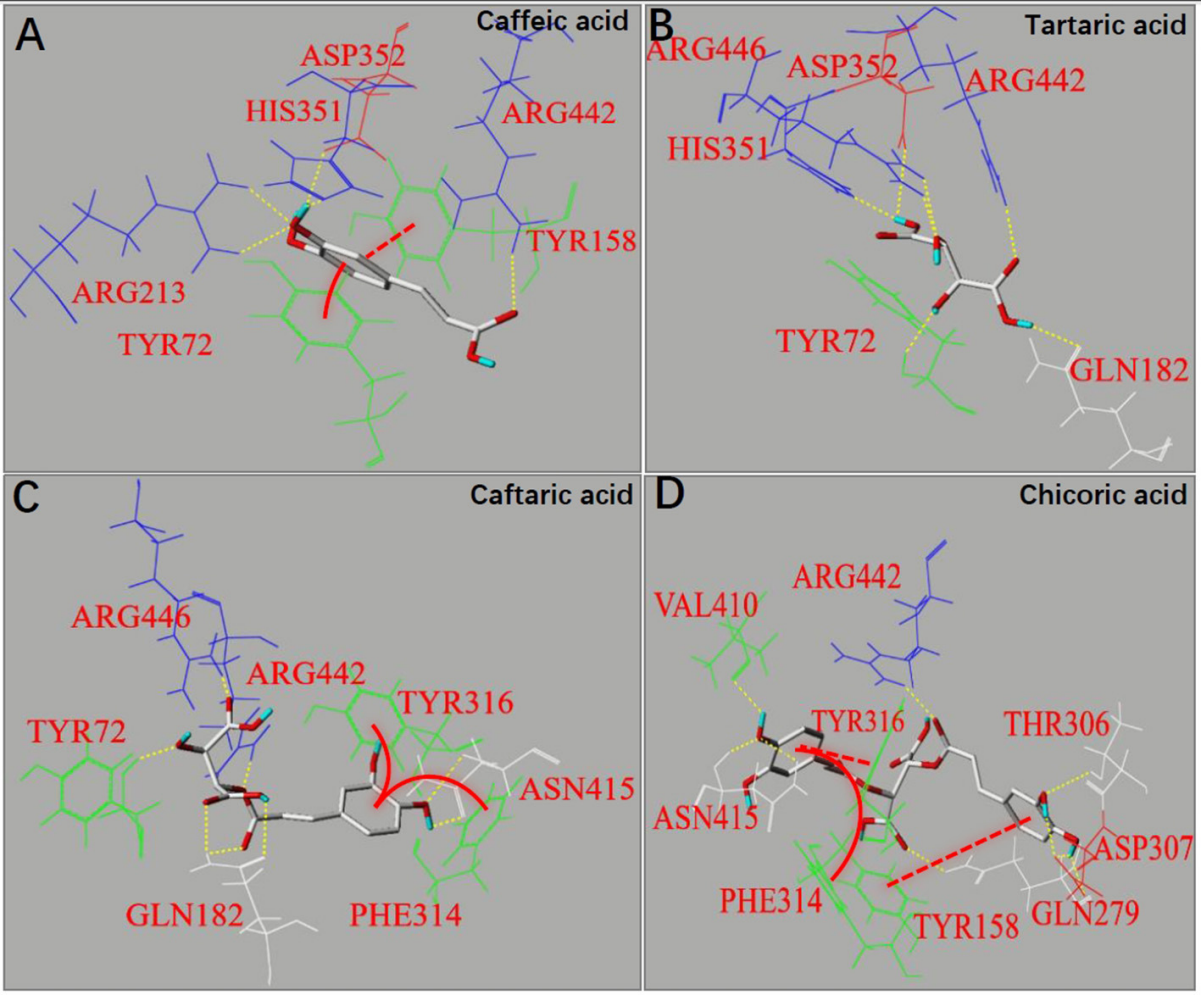
The molecular docking for binding interactions of caffeic acid **(A)**, tartaric acid **(B)**, caftaric acid **(C)** and chicoric acid **(D)** with α-glucosidase, respectively. The interaction forces involved hydrogen bonding (yellow dashed lines) and π-π hydrophobic forces, including parallel conjugations (red dashed lines) and vertical conjugations (red full curves).

Molecular docking is an effective approach to simulate binding interactions between a micromolecular ligand and a macromolecular protein at the active site, from which the interacting sites and non-covalent interaction forces can be obtained, as well as the binding efficiency (36). The docking method was used to study the molecular interactions between organic acids and α-glucosidase (Figure 5). It was found that π-π conjugations (stackings), including parallel and vertical ones were formed between the aromatic ring(s) of caffeoyl moiety(s) and that of fluorescent amino acids for CA, CFA and CHA, such as Try^72^, Tyr^158^, Phe^314^ or Tyr^316^ (Figures 5A,C,D), which confirms with the strong fluorescence quenching effect of the three organic acids (Figures 3A,C,D). Besides, one more caffeoyl moiety in CHA provided it with one more π-stacking with the enzyme (Figure 5D), causing the higher quenching effect than CA and CFA (Table 1). However, there was no π-conjugation of TA formed with α-glucosidase (Figure 5B) due to the lack of aromatic ring in TA; therefore, no fluorescence quenching was observed for this organic acid compound (Figure 3B).

As caffeoyl substitution at 2 and/or 3 -OH of TA (which keeps the carboxylic acid moieties intact, Figure 1A) significantly decreased the binding affinity and inhibitory activity of TA against α-glucosidase (Figures 1C, 4), the two -OH moieties played an important role in binding interactions with the enzyme, while the two carboxylic acid moieties mainly contributed to the acidic pH environment in solutions which finally caused the inactivation of the enzyme. Therefore, the non-covalent interaction forces involving the binding sites at 2 and/or 3 -OH of the organic acids mainly contributed to the reversible enzyme inhibition. It was observed that hydrogen bondings were formed between Arg^446^ and 2-OH (two bondings) and between Tyr^72^ and 3-OH (one bonding) in TA-glucosidase docking (Figure 5B). Both the amino acid residues of α-glucosidase have also been reported to take part in binding with micromolecular inhibitors (like polyphenols) (11, 12). Obviously, caffeoyl substitution at 2 and/or 3 -OH gradually disappeared the hydrogen bondings of the two -OHs with Arg^446^ and Tyr^72^ (Figures 5C,D), causing the decrease in inhibition effects of CFA and CHA compared with TA (Figure 1C).

As for the binding energy (*E*_b_) that indicates the binding efficiency of organic acids with the enzyme, it is interestingly found that along with the introduction of caffeoyl moiety(s), the *E*_b_ values of TA, CFA and CHA increased in sequence (Table 1). This is supposed to result from the increased interaction forces including hydrogen bondings (7 for CA, 8 for CFA, and 8 for CHA) and π-conjugations (0 for CA, 2 for CFA and 3 for CHA) due to the presence of caffeoyl moiety(s) (Figure 5). Even though, the increased non-covalent forces did not contribute to the inhibitory activity against α-glucosidase because of the disappearance of the essential hydrogen bondings regarding 2 and/or 3 -OH with Arg^446^ and Tyr^72^ as discussed above. On the other hand, the increased π-conjugations did increase the interactions of organic acids with the enzyme fluorophores, increasing the fluorescence quenching effects. Therefore, it is concluded that the inhibitory activity of an inhibitor against α-glucosidase is not necessarily related with its general interactions with the enzyme. Instead, it depends on the binding interactions (or binding affinity) of the inhibitor key moiety(s) with the essential amino acid residues that decide the catalytic activity of the enzyme.

## Conclusion

The inhibition of four organic acids against α-glucosidase was investigated in this study. Interestingly, although the four compounds, except for CA, were shown with different inhibition effects, there was a large gap of inhibition between two adjacent gradient organic acid concentrations and thus between two adjacent gradient pH values for each compound. Thus, the acidic environment at the relatively high organic acid concentrations tended to finally cause the acid-oriented inactivation of α-glucosidase. From the ITC result, only TA was shown with an obvious direct binding behavior with α-glucosidase. Therefore, the enzyme inhibition of CFA and CHA mainly resulted from the acidic pH values that were not suitable for the enzyme activity, while the inhibitory activity of TA was attributed to both the acidic pH and binding interactions with the enzyme. Besides, although caffeoyl moiety decreased the inhibitory activity of TA, the moiety provided the caffeoylated organic acids with a higher fluorescence quenching effect due to π-stacking between aromatic rings of caffeoyl and enzyme fluorophores, as well as with a higher docking efficiency. Therefore, the inhibitory activity of an inhibitor against α-glucosidase does not necessarily correspond to the interaction constants with the enzyme obtained from fluorescence quenching and molecular docking. Instead, it largely depends on the binding affinity of the inhibitor to the essential catalytic residues at the specific/active site of the enzyme.

## Data Availability Statement

The original contributions presented in the study are included in the article/supplementary material, further inquiries can be directed to the corresponding author.

## Author Contributions

WL: conceptualization, data curation, methodology, software, validation, and writing original draft. YS: methodology. WS: methodology and software. XY: methodology, software, and supervision. XL and LS: project administration, writing, review and editing, project administration, and fundings acquisition. All authors contributed to the article and approved the submitted version.

## Funding

This study is supported by the National Natural Science Foundation of China (No. 31901685).

## Conflict of Interest

The authors declare that the research was conducted in the absence of any commercial or financial relationships that could be construed as a potential conflict of interest.

## Publisher's Note

All claims expressed in this article are solely those of the authors and do not necessarily represent those of their affiliated organizations, or those of the publisher, the editors and the reviewers. Any product that may be evaluated in this article, or claim that may be made by its manufacturer, is not guaranteed or endorsed by the publisher.
